# Knowledge, Attitude, and Behavior about Antimicrobial Use and Resistance among Medical, Nursing and Pharmacy Students in Jordan: A Cross Sectional Study

**DOI:** 10.3390/antibiotics11111559

**Published:** 2022-11-05

**Authors:** Ghaith M. Al-Taani, Reema A. Karasneh, Sayer Al-Azzam, Maryam Bin Shaman, Feras Jirjees, Hala Al-Obaidi, Barbara R. Conway, Mamoon A. Aldeyab

**Affiliations:** 1 Department of Clinical Pharmacy and Pharmacy Practice, Faculty of Pharmacy, Yarmouk University, Irbid 21163, Jordan; 2 Department of Basic Medical Sciences, Faculty of Medicine, Yarmouk University, Irbid 21163, Jordan; 3 Department of Clinical Pharmacy, Faculty of Pharmacy, Jordan University of Science and Technology, Irbid 22110, Jordan; 4 Pharmacy Department, Prince Mohammad Medical City, Ministry of Health, Aljouf 72345, Saudi Arabia; 5 Department of Pharmacy Practice and Pharmacotherapeutics, College of Pharmacy, University of Sharjah, Sharjah P.O. Box 27272, United Arab Emirates; 6 College of Pharmacy and Health Sciences, Ajman University, Ajman P.O. Box 346, United Arab Emirates; 7 Department of Pharmacy, School of Applied Sciences, University of Huddersfield, Huddersfield HD1 3DH, UK; 8 Institute of Skin Integrity and Infection Prevention, University of Huddersfield, Huddersfield HD1 3DH, UK

**Keywords:** antimicrobial, education, medical, nursing, pharmacist, resistance, knowledge, behavior, prescribing practices, antimicrobial stewardship

## Abstract

The present study aimed to survey medical, nursing and pharmacy students’ knowledge, attitude and practice regarding antimicrobial use and resistance. Additionally, the study assessed the teaching and assessment activities received regarding antibiotic use. A cross sectional online survey was distributed to undergraduate students currently in clinical studies in their degree program. A total of 716 medicine, nursing and pharmacy undergraduate students were included. Respondents scored more than 76% on knowledge on effective use, unnecessary use and associated side effects of antibiotics, and 65.2% regarding knowledge on the spread of antibiotic resistance. Some participants (21.0%) agreed or strongly agreed that there has been good promotion of prudent antimicrobial use. Students were aware (13.1%), unaware (29.1%), or unsure (57.8%) that there is a national action plan relating to antimicrobial resistance. A total of 62.8% of the respondents strongly agreed or agreed that they have a key role in helping control antibiotic resistance. Participants reported that they require more information about resistance to antibiotics (53.9%), medical conditions for which antibiotics are used (51.7%) and how to use antibiotics (51.0%). Discussion of clinical cases and vignettes and small group teaching were reported as very useful or useful teaching strategies (79.9% and 74.2%, respectively). The findings from this study determined the current situation in relation to education on prudent antimicrobial use for undergraduates and highlighted areas for informing better curriculum design.

## 1. Introduction

Antimicrobial resistance has a global impact on public health, leading to increased morbidity, mortality and healthcare costs [1,2,3,4]. Antibiotics, as a group of medications, have been subject to indiscriminate use [5], which is considered to be a primary factor in the development of antimicrobial-resistant microorganisms, leading to complications in patients [5,6,7,8,9]. Healthcare professionals are directly involved in the prescribing and use of antibiotics. They might respond to patients’ inappropriate requests or might carry out inappropriate practices, which in turn increase the chances for antimicrobial resistance [10,11]. On the contrary, awareness and information provision related to prudent antimicrobial use could inform better decisions by healthcare professionals [12,13]. Awareness of initiatives addressing antimicrobial resistance is very important and should be directed toward healthcare professionals, students and patients. As patients are responsible for taking their medicines while they are not in a healthcare setting, inappropriate behavior by patients and deficiencies in knowledge can negatively impact antimicrobial resistance [14]. In order to retain the efficacy of antibiotics, antimicrobial stewardship (AMS) was developed to address the inappropriate prescribing and utilization of antimicrobials and to mitigate the risk of antimicrobial resistance [15]. 

In an effort to tackle the problem of antimicrobial resistance, education of the new generation of healthcare professionals should ensure that medical and health students develop the competency required to provide effective and safe quality patient care [16]. There has been an increased emphasis on active teaching strategies that allow for deep learning [17]. Healthcare professionals are expected to encounter patients in practice, often on their own.As such, simulated and actual patient care practices need to be offered in the educational setting as they proceed in their degree program [18]. This includes embedding clinical concepts into the student educational experience, as well as using well-designed activities such as clinical placement, role play, introductory and advanced patient care experiences [19]. 

It is important to assess the collegial aspect of healthcare. This approach could be helpful in improving attitudes of future healthcare professionals toward prudent antimicrobial use [20]. Additionally, this approach could bring an awareness of the roles of other healthcare professionals and provide insight into acting within professional boundaries [21]. AMS programs require collaboration from many healthcare professionals and local healthcare organizations and hospitals [22,23]. 

It has been reported that medical and pharmacy students do not receive adequate antimicrobial management and stewardship training in the early years of study—and the effectiveness of the education is unknown [20,24]. It is worth mentioning that relatively limited literature is available that focuses on antimicrobial stewardship in undergraduate study, particularly targeting medicine, nursing and pharmacy students. What does exist can be restricted to certain geographical locations [25,26]. A study from Nigeria found that healthcare students had moderate knowledge of antimicrobial resistance and highlighted the need for better educational activities [27]. Findings were similar for Polish medical students [28]. It was reported that undergraduate pharmacy students from Zambia, despite demonstrating acceptable knowledge and attitudes towards antimicrobial use and resistance, still warranted an improvement in practice [29]. Two studies from Jordan assessed the knowledge, attitude and behavior of medical and nonmedical students as they related to antibiotic use and resistance; however, details regarding enrollment were not included [30,31] Pharmacy students in Jordan were surveyed and reported adequate knowledge, attitude and practice toward antibiotic use [32].

The present study aimed to survey medical, nursing and pharmacy students’ knowledge, attitude and behavior regarding antimicrobial use and resistance in Jordan using an adapted version of the European Centre for Disease Prevention and Control (ECDC) instrument [33,34]. A secondary aim was to assess teaching and assessment strategies these students were exposed to during their degree program. 

## 2. Results

### 2.1. Demographic Characteristics

A total of 716 undergraduate students studying in the medicine, nursing and pharmacy schools were recruited. Only those who were in their third year or above in their degree program for medicine and nursing students (and fifth year or more for the pharmacy students) were retained. The majority of the recruited students were between the ages of 18–25 years, with more than half of the participants being female (56.8%). The demographic characteristics and distribution of students in the three schools are shown in Table 1. 

### 2.2. Information Resources Used for Management of Infections

Students utilized social media networks for professional activities; the most common network used was Facebook^®^ (72.7%). Other resources utilized for the management of infections included high-quality resources, such as continuing education training courses (40.1%), and low-quality sources, such as social media (18%). Interestingly, few students utilized scientific journals as a source of information (4.2%) and 18% of the respondents had used social media. Details regarding these sources are summarized in Table 2.

### 2.3. Capabilities, Opportunities, Motivation and Behavior of Student Healthcare Professionals Regarding Antibiotic Use and Resistance

In relation to capabilities of student healthcare professionals, actual knowledge regarding antibiotic use and resistance was confirmed in many questions (i.e., percentage of correct key knowledge questions above 75%, such as efficacy of antibiotics against viruses or cold and flu, unnecessary use of antibiotics making them ineffective, and side effects associated with antibiotics). In contrast, few respondents (17%) answered the key knowledge question related to antibiotics and environment correctly. Details regarding the key knowledge question and responses are summarized in Table 3. 

Table 4 summarizes perceived knowledge, opportunity and motivation related to prudent antimicrobial use. The respondents scored well in many items related to perceived knowledge regarding issues related to prudent antimicrobial use, such as “I know what antibiotic resistance is” and “I know what information to give to individuals about the prudent use of antibiotics and antibiotic resistance”. Respondents, however, scored lower (<17%) in perceived knowledge related to antibiotics and the environment. In relation to opportunity, fair responses were obtained for this scale, i.e., about half of the responses agreed or strongly agreed that they had easy access to guideline and educational material to provide advice on prudent antibiotic use and antibiotic resistance in patients. Respondent students believed that they could mitigate the risk of antimicrobial resistance; for example, 62.8% of the responses strongly agreed or agreed that they had a key role in helping control antibiotic resistance. In relation to behavior, Table 5 illustrates that there was low provision of advice given to individual patients by students and even lower provision of resources to individual patients. 

### 2.4. Awareness of Initiatives That Are Focused on Antibiotic Use

In relation to national policy initiatives which focused on antimicrobial resistance, respondents stated that actions at all levels (i.e., individual, environmental and regional/national) are required to tackle resistance to antibiotics. To appropriately protect the public from the negative effects of antibiotic resistance, public health agencies should take an active role in raising awareness of initiatives to address antimicrobial resistance. As future healthcare professionals, more than a third (34.6%) of the surveyed students were not aware of any initiatives focused on antibiotic awareness and resistance. The most common initiatives (27.1%) involved awareness arising from professional organizations. Only 21.6% of the participants agreed or strongly agreed that there had been strong promotion of prudent antimicrobial use. In addition, just 13.1% of the students were aware that there was a national action plan on antimicrobial resistance. Table 6 shows a summary of initiatives at the national level that have focused on antimicrobial resistance. 

### 2.5. Education and Assessment Activities Related to Antibiotic Use and Resistance 

Table 7, Table 8 and Table 9 summarize the assessment of educational approaches received by the students during their undergraduate study period. More than three quarters of the students received teaching or examinations about prudent antibiotic use and management of infections. They reported that they most commonly required more information about resistance to antibiotics (53.9%), medical conditions for which antibiotics are used (51.7%) and how to use antibiotics (51.0%). Regarding the teaching strategies used, most found discussion of clinical cases and vignettes very useful or useful (79.9%), along with small group teaching (74.2%). A total of 67% viewed infectious disease clinical placements as very useful or useful, and 56% viewed role play or communication skills sessions dealing with patients demanding antibiotic treatment in a positive light. 

## 3. Discussion

Educating the next generation of health care professionals is a strategic goal. As antimicrobial resistance is a global threat and healthcare professionals are involved with prescribing, dispensing, administering and using antibiotics, promoting prudent antimicrobial use is a major quality and safety requirement to mitigate the risk of antimicrobial resistance. This is coupled with the importance of educating patients and the public about appropriate practices regarding infection control, use of antimicrobials and the effect of antibiotic use on the environment. Therefore, it is imperative to ascertain whether institutions are effectively educating medical, nursing and pharmacy students about appropriate antimicrobial use in an effort to modify the impact that antimicrobial resistance has on patient health. A well-known model for research of this type is the capabilities, opportunities, motivation and behavior survey, usually referred to as COM-B [35]. Of interest, the present study utilized the COM-B model to ascertain medical, nursing and pharmacy students’ knowledge, attitude and behavior toward a more prudent approach for antimicrobial use. This was first carried out in the Middle East utilizing a rigorous approach developed by the European Centre for Disease Prevention and Control (ECDC) [33,34]. The present study revealed good actual and perceived knowledge and fair motivation related to antimicrobial use and resistance. Limitations were noted in relation to opportunity, i.e., access to guidelines and education materials, behavior and awareness of the national action plan on antimicrobial resistance. Furthermore, the present study attempted to directly audit the type of teaching, assessment and evaluation methods related to antimicrobial use and resistance provided to students in their undergraduate studies, as reported by the students themselves. These endeavors are expected to add to the research literature and could serve as a base to enhance future educational strategies. 

### 3.1. Capabilities, Opportunities, Motivation and Behavior of Student Healthcare Professionals Regarding Antibiotic Use and Resistance

In accordance with the ECDC survey employing the COM-B model, the capabilities that were surveyed in the present study highlighted that there was good actual knowledge of many items related to prudent antimicrobial use. These topics were commonly covered in curricula, such as antibiotics ineffectiveness against viruses (e.g., cold and flu), and that unnecessary use of antibiotics leads to them becoming ineffective. However, some items, such as those related to the effect of antibiotics on the environment, were less well known and understood. Similar poor knowledge of the effect of antibiotics in agriculture was reported by healthcare professional students from Sri Lanka, while good knowledge was expressed by students from UK and moderate knowledge was reported from pharmacy students from Trinidad and Tobago and India [36,37,38]. In another study in Colombia, healthcare students reported good knowledge regarding antibiotic use and resistance. In India, 85% of students had correct knowledge on statements concerning inappropriate use of antibiotics that might lead to antimicrobial resistance [39,40]. However, in research conducted in Indonesia, Malaysia, and Pakistan, poor knowledge was demonstrated by healthcare students regarding statements related to the negative effects of antibiotic resistance and circumstances in which no treatment with an antibiotic was warranted [41]. In the UK, pharmacy students scored well in the knowledge scales about antibiotic use and resistance [37]. Knowledge related to antibiotic use and resistance would enable them to apply prudent antimicrobial use principles [42]. Healthcare professional knowledge could be imparted from different resources, such degree program study (which was the focus of the present study), continuous education, preregistration training, work experience and the full range of lay and professional information resources available [43]. An important gatekeeper is the training healthcare professionals receive in their education, in which the efficacy of the teaching of antimicrobials has been widely reported [25], as there is a possibility that any knowledge gained will not be translated into practice [44]. 

High perceived knowledge was communicated by the surveyed students, which could have been related to positive self-impressions regarding antibiotic use. However, lower scores were achieved for perceived knowledge relating to the effects of antibiotics on the environment. Perceived knowledge is considered an integral part of the COM-B model and influences behavior [33,34]. During their degree program, students should be prepared for their future professions through useful educational experiences. Medical students surveyed in a previous study highlighted that they had paid attention to antimicrobial resistance caused by inappropriate antimicrobial use and requested action to improve prescribing practices in the future [45]. Others highlighted that pharmacy students from India reported a poor attitude toward antibiotic use and resistance [40]. It has become necessary to enhance the knowledge of pharmacy students and other healthcare professionals regarding appropriate antimicrobial use in order to equip them with the required skills to address practice challenges [46,47,48,49,50]. Data from the UK indicated that about 50% of surveyed pharmacy students in the third and fourth years of their M Pharm degree reported that they had acquired the skills they needed to practice appropriate antibiotic use from their degree program—highlighting the wide variations in the design of educational programs between countries [37]. 

About half of the respondents in the present study reported that they had the opportunity to address antimicrobial use, in terms of easy access to guidelines and educational material for antibiotics, and had opportunities to provide advice on prudent antibiotic use. This highlighted that the professional work environment and resources were conducive to the application of initiatives to promote prudent antimicrobial use. However, a fair level (<70%) was reported for items related to access to guidelines and advice material and provision of advice. Healthcare professionals play a pivotal role in the management of antimicrobial use, e.g., prescribing, dispensing and administration of antimicrobial agents, patient education regarding appropriate use of antimicrobial agents and against antimicrobial self-medication. If these aspects are not delivered appropriately, this will increase the chance of antimicrobial resistance [40]. There was a variation in attitude between healthcare professional students regarding antimicrobial use and resistance, e.g., a good attitude was reported by students from Columbia, whereas the attitude of students from India showed room for improvement [39,40]. Students should be offered more support to address this limitation, as the provision of appropriate, well-designed curricula for students addressing antimicrobial stewardship has been linked with graduating healthcare professionals who feel confident with their abilities to support prudent antimicrobial use [51]. 

In relation to the motivation domain, many students believed that they had a key role in addressing the issue of antimicrobial resistance. This could be interpreted as reflecting on high self-confidence in this regard. Antimicrobial resistance can be a result of poor antimicrobial knowledge and perception [52,53], while provision of educational interventions can positively influence attitudes concerning antibiotic use and resistance [54,55]. In some surveys, healthcare professional students demonstrated that they had a good attitude toward antibiotic use and resistance [56]. Patient care processes, or behaviors related to prudent antimicrobial use, were carried out frequently by respondent students, which confirmed that appropriate information was provided within the curricula. Despite this, it was unclear whether this could positively impact the issue of antimicrobial resistance in the future [57]. It has also been reported that healthcare students might have the knowledge but have a problem regarding appropriate attitudes, such as facilitating self-medication with antibiotics despite having correct knowledge concerning antimicrobial resistance. This highlighted that educational institutions should try to improve the attitude of their students toward antimicrobial stewardship [40,52,58,59]. It is well-known that, with improved training, better behavior can be achieved [60]. In spite of this, changing the habits of healthcare professionals is difficult [61,62], and the most-studied approach to influence habits in antimicrobial stewardship is education [22,63]. More information, particularly within practice, is needed to gain insights into routine practices in relation to prudent antimicrobial use, taking into consideration the level of experience and the baseline beliefs of the healthcare professional [64]. Many authors have highlighted the need for greater emphasis in undergraduate healthcare professional curricula on topics related to antimicrobial stewardship [65,66,67].

### 3.2. Awareness of Initiatives That Are Focused on Antibiotic Use

With alarming figures of antimicrobial resistance, rational antimicrobial use is needed [68]. Governmental policies and public health campaigns play a key role in addressing the issue of antimicrobial resistance and promoting prudent antimicrobial use. Many initiatives exist, including some at the healthcare policy level, such as awareness and infection control practices—an exemplar being the WHO global action plan focusing on enhancing awareness of antimicrobial resistance [36]. Surveyed students in the present study were commonly aware of such initiatives from professional organizations and TV or radio advertising for the public, which indicated utilization of the media as a way to influence the public about antimicrobial issues. Topics related to antimicrobial stewardship have ben reported in the media, indicating the role of the public in addressing this issue [39,40,52]. Similar campaigns have been planned, directed toward lay public and healthcare professionals, to raise awareness of issues related to antibiotic use and resistance [14,69,70]. A high percentage (83%) of healthcare professional students from Rwanda were unaware of the concept of antimicrobial stewardship and it was not even covered in the curriculum, as reported by almost half of the respondents, which highlighted this issue in some developing countries [71]. Other researchers found that healthcare professional students from India were aware of antimicrobial stewardship [40]. As such, it has been reported that it is important to raise the awareness of antimicrobial stewardship among prescribers [72]. About 40% of the students in the present study reported that action was required to address resistance to antibiotics in all levels, including prescribers and other healthcare professionals, on the national level. Such results were challenged by the negative perception of students in the present study about promotion of prudent use of antibiotics and antibiotic resistance in their country and pronounced low awareness of the national action plan on antimicrobial resistance [73]. Healthcare professional students in previous research reported that it was important to undertake large-scale campaigns to address concerns related to antibiotic use and resistance [56,74]. In Jordan, an action plan on antimicrobial resistance was developed, and was endorsed by the World Health Organization, yet awareness and uptake seemed to fall below the expected level. Similarly, less than 50% of pharmacy students surveyed, including PharmD students, were unaware of the national antibiotic policy in India [46]. 

### 3.3. Education and Assessment Activities Related to Antibiotic Use and Resistance

Taking into consideration that educating healthcare professionals is not a straightforward task, continuous audits and assessments should be made. As antimicrobial stewardship education has not always been not included at early stages within undergraduate training, there have been gaps in curricula regarding antimicrobial use [20]. Shortcomings in the educational process have been reported, such as disagreements between microbiology course work and clinical practice and that routine clinical procedures undertaken in practice can hinder prudent antimicrobial prescribing [75,76,77]. The present study found that a high percentage of students received teaching and assessment for prudent antibiotic use and management of infection and considered that their educational methods were very useful or useful. This could mean that the education of future healthcare professionals will employ active learning, invest in eLearning platforms and continue to utilize traditional techniques such as lecture and case discussion. In a survey of healthcare professional students in Rwanda, it was reported that >60% of the students discussed antimicrobial resistance or received a course about antimicrobial resistance during their degree course [71]. The WHO, Infectious Disease Society of America, Society of Healthcare Epidemiology of America and Pediatric Infectious Disease Society have recommended that the undergraduate curricula for medical schools should include training on antimicrobial stewardship [23,78,79,80,81,82,83]. Others highlighted the need for increased emphasis on antimicrobial stewardship in undergraduate curricula [65,66,67,84,85]. Active learning strategies, however, are more commonly employed in postgraduate studies [57]. Despite some educational elements about antimicrobial stewardship being present in the curricula, information regarding the efficiency of the education strategies for all levels was lacking [57] and it seemed that there was a low level of preparedness and confidence of healthcare professional students in relation to antimicrobial stewardship topics in their undergraduate studies [86]. The Infectious Diseases Society of America developed an antimicrobial stewardship curriculum for pharmacy and medical students. It included clinical vignettes; the approach was reported to increase students’ application of antimicrobial stewardship approaches and foster interprofessional collaboration [20]

The students surveyed reported that they needed more information about several topics related to antimicrobial stewardship, such as resistance to antibiotics. With an increased abundance of information resources, it is important to ascertain what resources are available for healthcare professionals and how often they are utilized. Coupled with this, many of the health disciplines studied are ever-changing, so there is a need to prepare the graduate as a lifelong learner and ensure that healthcare professionals’ knowledge is up-to-date. Interestingly, the present study highlighted that social media platforms have been utilized to a high degree for professional activities by the surveyed students. Such resources’ quality of information is questionable; however, they can provide timely information in an interactive manner. To put things into perspective: despite criticism regarding the quality of information resources available online, particularly healthcare resources, healthcare technology is a fact of life nowadays. The overwhelming majority of people in the world have online access. It follows logically that we should be raising awareness of quality issues in online healthcare resources for students, healthcare professionals and patients. Pharmacy students in Indonesia, Malaysia, and Pakistan relied on the internet and degree courses to obtain health information [41]. From the professional information resources that the students used routinely, the most common were continuing education training courses and previous clinical experience. It was surprising that scientific journal resources were used by only 4.2% of the respondents, possibly highlighting an underrepresentation of scientific journals in educational curricula. Overall, information resource provision has been viewed as a major factor that could influence behavior. This would be very helpful in efforts to change the attitude of healthcare professionals and students, especially in the antimicrobial stewardship initiative [54]. 

There is also a need to improve the content, teaching approach and assessment of healthcare professionals’ curricula on issues relating to prudent antimicrobial use and antimicrobial stewardship [25,51,87]. To realize these, modern active teaching and assessment techniques should be utilized. Areas of interest to support prudent antimicrobial use include microbiology, clinical pharmacology, infectious disease, and antimicrobial stewardship [37]. Antimicrobial stewardship training is recommended to be undertaken early in the degree program, including an interdisciplinary element. Further research is recommended to ascertain the factors involved in the development of inappropriate antimicrobial use by physicians and other healthcare professionals, such as prescribing research and think-aloud studies. The study had some limitations. Due to the online distribution of the instrument, there was a possibility of selection bias to those with access to the internet. However, nowadays, availability of information technology and smartphones are widespread among students. 

In conclusion, the present study aimed to assess medical, nursing, and pharmacy students’ preparedness for behavior change toward prudent antimicrobial use and related educational instruction provided for those students in Jordan. It was noted that the respondent students had good actual and perceived knowledge regarding antimicrobial use and resistant issues surveyed in the present study, coupled with fair motivation, i.e., reporting that they had a key role in tackling antimicrobial resistance. They reported lower scores for opportunity, concerned with easy access to guidelines and material to address antimicrobial resistance, and behavior, represented by practices carried out to address antimicrobial resistance. Although student healthcare professionals were knowledgeable and had a positive attitude toward antibiotic use and resistance, there wer limitations in opportunity and behavior regarding prudent antimicrobial use and low awareness of the national action plan for antimicrobial resistance. Better curriculum design will be required to address the limitations noted in the present study.

## 4. Methods

A survey was developed to assess the knowledge, attitude and practice of medical, nursing and pharmacy students in the years in their degree program in which they received clinical teaching in Jordan relating to antibiotic use and resistance, utilizing an adapted version of the European Center for Disease Prevention and Control (ECDC) capabilities, opportunities, motivation and behavior (COM-B) model [33,34]. The questionnaire was distributed online using the survey administration software Google Forms. Students in medicine, nursing and pharmacy schools across all universities in Jordan (approximately 25,000 students) were targeted. The selection criteria included medicine students in the third, fourth or fifth year or more, nursing students in the third and fourth year and pharmacy students (BPharm and PharmD) in the fifth year or above. These criteria facilitated better targeting to those who had practical experience of their future profession in practice sites in primary and secondary care. Graduates, students not in the targeted years, students studying outside Jordan and those studying other disciplines were excluded from the study. 

Eligible students were provided with an invitation to complete the survey via the web, which included a link to the Google Form. The sampling framework included students’ email addresses and social media professional pages. To ensure compliance with ethical standards, the invitation described the study and what it involved, the voluntary nature of the study and the anonymous distribution of the survey, i.e., no identifiers that could link to the participants or their location were collected. Only the participants who agreed to take part in the survey had access to the survey, as an accept option was embedded in the first page of the survey and those who did not agree to take part were directed out of the Google Form. The settings for the form applied included required options and options limited to one response, in order to increase response quality and efficiency. 

An adapted version of a European Center for Disease Prevention and Control (ECDC) validated instrument was utilized in the present study to fulfill the study objectives [33,34]. The respondents had the option to complete the survey in either English or Arabic. The research team rechecked the content and face validity of the adapted instrument, via review by faculty members with expertise in the field and postgraduate degrees. Based on their comments, minor changes were made in the instrument. The Arabic version of the instrument was subjected to a forward-backward procedure, as well. The survey was pilot-distributed to 10 students to assess the clarity of the questionnaire and distribution logistics; comments received were taken into consideration in the development in the questionnaire. The final analysis did not include the data from the pilot distributed. 

The Supplementary Material includes the instrument used (Supplementary Material). The 46-item instrument consisted of a number of sections concerning knowledge, attitude and behavior relating to antibiotic use and resistance, and educational techniques used during their study for the degree program regarding the same. The first section asked about the demographics of the respondents, i.e., age and gender, their respective faculty and year of study, and information resources used regularly for issues related to antibiotic use and resistance. Additionally, the survey asked about capabilities, in terms of actual and perceived knowledge, the opportunity factors that prompted behavior, the motivation to carry out behavior, and the behavior itself. Furthermore, collected data regarding the public health strategies were made available to tackle the antimicrobial resistance. The last section included an assessment of the teaching strategies and assessment students were exposed to during their degree program regarding antibiotic use and resistance, together with reported outcomes. 

Sample size calculations: With an estimated number of medical, nursing and pharmacy students of 25,000 students, a 5% margin of error and a 95% confidence interval, the calculated sample size need was estimated to be 379, according to online Raosoft sample size calculator (http://www.raosoft.com/samplesize.html; accessed on 13 September 2022). Statistical Package for the Social Sciences (SPSS) IBM (version 24) was used to run the analysis. Descriptive statistics were used to describe trends within the data. 

## Figures and Tables

**Table 1:** Demographic characteristics of medical, nursing and pharmacy students (*n* = 716).

**Demographic Characteristics**				
			***n* (%)**	
Age (years)		18–25	663 (92.6%)	
		26–35	47 (6.6%)	
		36–45	6 (0.8%)	
Sex		Male	309 (43.2%)	
		Female	407 (56.8%)	
**School and Year of Study**				
		Medicine *n* (%)	Nursing *n* (%)	Pharmacy *n* (%)
Year of study	Third	74 (18.3)	49 (46.2)	0 (0.0)
	Fourth	110 (27.2)	57 (53.8)	0 (0.0)
	Five or more	221 (54.6)	0 (0.0)	205 (100.0)
	Total	405 (56.6)	106 (14.8)	205 (28.6)

**Table 2:** Social media networks used by the students for professional activities and the information resources used by the students regularly in the management of infections (*n* = 716).

**Social Media Networks Used by the Students for Professional Activities**	
	***n* (%)**
Facebook	520 (73%)
Instagram	178 (25%)
YouTube	165 (23%)
Google	107 (15.0%)
Twitter	62 (9%)
LinkedIn	42 (6%)
Google+	31 (4%)
I do not use social media	38 (5%)
Others (Email, WhatsApp., and/or Telegram)	5 (1%)
**Information Resources Used by the Students Regularly in the Management of Infections**	
	***n* (%)**
Medical representatives	31(4.0)
Previous clinical experience	207 (29.0)
Continuing education training courses	287 (40.0)
Infection specialists	62 (9.0)
Scientific journal	30 (4.0)
Professional resources	117 (16.0)
Social media	129 (18.0)
Others	27 (4.0)
I don’t know	55 (8.0)

**Table 3:** Actual knowledge of respondent students related to antibiotic use and resistance.

Questions Assessed the Knowledge Level	Correct Answer	Answer	*n* (%)
Antibiotics are effective against viruses	False	False	612 (85.5)
		True	81 (11.3)
		Unsure	23 (3.2)
Antibiotics are effective against cold and flu	False	False	549 (76.7)
		True	133 (18.6)
		Unsure	34 (4.7)
Unnecessary use of antibiotics makes them become ineffective	True	False	32 (4.5)
		True	666 (93.0)
		Unsure	18 (2.5)
Taking antibiotics has associated side effects or risks such as diarrhea, colitis, allergies	True	False	28 (3.9)
		True	618 (86.3)
		Unsure	70 (9.8)
Every person treated with antibiotics is at an increased risk of antibiotic resistant infection	True	False	121 (16.9)
		True	521 (72.8)
		Unsure	74 (10.3)
Antibiotic resistant bacteria can spread from person to person	True	False	126 (17.6)
		True	467 (65.2)
		Unsure	123 (17.2)
Healthy people can carry antibiotic resistant bacteria	True	False	61 (8.5)
		True	478 (66.8)
		Unsure	177 (24.7)
The use of antibiotics to stimulate growth in farm animals is legal in Jordan	False	False	124 (17.3)
		True	80 (11.2)
		Unsure	512 (71.5)

**Table 4:** Perceived knowledge, opportunity and motivation of respondent students regarding antibiotic use and resistance (*n* = 716).

Item	SA *n* (%)	A *n* (%)	D *n* (%)	SD *n* (%)	N/A *n* (%)	U *n* (%)	IDU *n* (%)
Perceived knowledge							
I know what antibiotic resistance is	264 (36.9)	351 (49.0)	16 (2.2)	16 (2.2)	3 (0.4)	60 (8.4)	6 (0.8)
I know what information to give to individuals about the prudent use of antibiotics and antibiotic resistance	95 (13.3)	379 (52.9)	57 (8.0)	11 (1.5)	15 (2.1)	151 (21.1)	8 (1.1)
I have sufficient knowledge about how to use antibiotics appropriately for my current practice	77 (10.8)	344 (48.0)	84 (11.7)	15 (2.1)	19 (2.7)	165 (23.0)	12 (1.7)
Environmental factors such as waste water in the environment are important in contributing to antibiotic resistance in bacteria from humans	40 (5.6)	244 (34.1)	76 (10.6)	19 (2.7)	-	251 (35.1)	86 (12.0)
Excessive use of antibiotics in livestock and food production is important in contributing to antibiotic resistance in bacteria from humans	124 (17.3)	306 (42.7)	32 (4.5)	16 (2.2)	-	186 (26.0)	52 (7.3)
Opportunity							
I have easy access to guidelines I need on managing infections	64 (8.9)	317 (44.3)	114 (15.9)	24 (3.4)	30 (4.2)	155 (21.6)	12 (1.7)
I have easy access to the materials I need to give advice on prudent antibiotic use and antibiotic resistance	53 (7.4)	269 (37.6)	146 (20.4)	33 (4.6)	34 (4.7)	175 (24.4)	6 (0.8)
I have good opportunities to provide advice on prudent antibiotic use to individuals	63 (8.8)	332 (46.4)	92 (12.8)	22 (3.1)	35 (4.9)	165 (23.0)	7 (1.0)
Motivation							
I know there is a connection between my prescribing OR dispensing OR administering of antibiotics and emergence and spread of antibiotic resistant bacteria	206 (28.8)	309 (43.2)	26 (3.6)	18 (2.5)	53 (7.4)	79 (11.0)	25 (3.5)
I have a key role in helping control antibiotic resistance	132 (18.4)	318 (44.4)	55 (7.7)	20 (2.8)	32 (4.5)	147 (20.5)	12 (1.7)

SA: Strong agree, A: Agree, D: Disagree, SD: Strong disagree, N/A: Not applicable, U: Undecided, IDU: I don’t understand the question.

**Table 5:** Behavior regarding antimicrobial resistance reported by the respondent students.

Frequency of Provision	Resources on Prudent Antibiotic Use Were Given to Individuals during the Last One Week? *n* (%)	Advice Related to Prudent Antibiotic Use Were Given to an Individual during the Last One Week *n* (%)
More than once a day	18 (2.5)	37 (5.2)
once daily	15 (2.1)	29 (4.1)
More than once a week	39 (5.4)	90 (12.6)
Once a week	54 (7.5)	110 (15.4)
Rarely	158 (22.1)	167 (23.3)
Never	239 (33.4)	124 (17.3)
Not applicable	114 (15.9)	80 (11.2)
I don’t remember	79 (11.0)	79 (11.0)

**Table 6:** National policies and initiatives that are focused on antimicrobial resistance.

**Variable**	**Categories**					***n* (%)**	
The level most effective to tackle resistance to antibiotics	Individual level (prescribers)					302 (42.2)	
	Environmental/animal health					56 (7.8)	
	Regional/national level					87 (12.2)	
	Action at all levels					287 (40.1)	
	I do not know					52 (7.3)	
Initiatives you are aware of in your country which focus on antibiotic awareness and resistance	TV or Radio advertising for the public					168 (23.5)	
	Toolkits and resources for healthcare workers					144 (20.1)	
	National or regional guidelines on management of infections					107 (14.9)	
	Awareness raising from professional organizations					194 (27.1)	
	Conference/Events focused on tackling antibiotic resistance					104 (14.5)	
	National or regional posters or leaflets on antibiotic awareness					89 (12.4)	
	Newspaper (national) articles on antibiotic resistance					42 (5.9)	
	World Antibiotic Awareness Week					35 (4.9)	
	I am not aware of any initiatives					248 (34.6)	
	Other					5 (0.7)	
**Perception about promotion of prudent use of antibiotic and antibiotic resistance**							
**Variable**	**SA** ***n* (%)**	**A** ***n* (%)**	**D** ***n (*%)**	**SD** ***n* (%)**	**N/A** ***n* (%)**	**U** ***n* (%)**	**IDU** ***n* (%)**
There has been good promotion of prudent use of antibiotics and antibiotic resistance in my country	28 (3.9)	127 (17.7)	162 (22.6)	64 (8.9)	220 (30.7)	69 (9.6)	46 (6.4)
**Awareness for national action plan on antimicrobial resistance**							
**Variable**	**No** ***n* (%)**		**Yes** ***n* (%)**		**Unsure** ***n* (%)**		
Does your country have a national action plan on antimicrobial resistance	208 (29.1)		94 (13.1)		414 (57.8)		

SA: Strong agree, A: Agree, D: Disagree, SD: Strong disagree, N/A: Not applicable, U: Undecided, IDU: I don’t understand the question.

**Table 7:** Teaching received by the students regarding antibiotic use.

Variable	No *n* (%)	Yes *n* (%)	Unsure *n* (%)
Had teaching about prudent antibiotic use	55 (7.7)	620 (86.6)	41 (5.7)
Had teaching about management of infections (diagnosis and antibiotic treatment)	54 (7.5)	630 (88.0)	32 (4.5)
Had examinations about prudent antibiotic use	99 (13.8)	547 (76.4)	70 (9.8)
Had examinations about management of infections (diagnosis and antibiotic treatment)	75 (10.5)	591 (82.5)	50 (7.0)

**Table 8:** Educational needs assessment in relation to antibiotic use and resistance.

Variables		*n* (%)
Topics you would like to receive more information about	Resistance to antibiotics	386 (53.9)
	How to use antibiotics	365 (51.0)
	Medical conditions for which antibiotics are used	370 (51.7)
	Prescription of antibiotics	338 (47.2)
	Links between the health of humans, animals and the environment	194 (27.1)
	none	44 (6.1)

**Table 9:** Outcome of teaching within undergraduate study regarding antibiotic use.

Teaching Method	Very Useful *n* (%)	Useful *n* (%)	Not Useful *n* (%)	Not Very Useful *n* (%)	N/A *n* (%)	Undecided *n* (%)	IDU *n* (%)
Lectures (with >15 people)	102 (14.2)	400 (55.9)	49 (6.8)	26 (3.6)	33 (4.6)	100 (14.0)	6 (0.8)
Small group teaching (with <15 people)	242 (33.8)	289 (40.4)	10 (1.4)	11 (1.5)	110 (15.4)	48 (6.7)	6 (0.8)
Discussions of clinical cases and vignettes	298 (41.6)	273 (38.1)	7 (1.0)	11 (1.5)	68 (9.5)	47 (6.6)	12 (1.7)
Active learning assignments (e.g., article reading, group work, preparing an oral presentation)	171 (23.9)	314 (43.9)	34 (4.7)	20 (2.8)	88 (12.3)	86 (12.0)	3 (0.4)
E-learning	62 (8.7)	227 (31.7)	118 (16.5)	100 (14.0)	34 (4.7)	167 (23.3)	8 (1.1)
Role play or communication skills sessions dealing with patients demanding antibiotic training	120 (16.8)	281 (39.2)	23 (3.2)	14 (2.0)	168 (23.5)	100 (14.0)	10 (1.4)
Infectious diseases clinical placement (i.e., clinical rotation or training in infectious diseases, involving patients)	240 (33.5)	240 (33.5)	16 (2.2)	9 (1.3)	143 (20.0)	60 (8.4)	8 (1.1)
Microbiology clinical placement	133 (18.6)	264 (36.9)	19 (2.7)	4 (0.6)	177 (24.7)	105 (14.7)	14 (2.0)
Peer or near-peer teaching (i.e., teaching led by other students)	128 (17.9)	342 (47.8)	32 (4.5)	4 (0.6)	107 (14.9)	96 (13.4)	7 (1.0)

N/A: Not applicable, IDU: I don’t understand the question.

## Data Availability

All collected data for this study are published in this article.

## Supplementary Materials

The following supporting information can be downloaded at: https://www.mdpi.com/article/10.3390/antibiotics11111559/s1, Supplementary Material: Survey of students of health sciences knowledge and attitudes about antibiotics and antibiotic resistance

## References

[1] Murray C.J., Ikuta K.S., Sharara F., Swetschinski L., Robles Aguilar G., Gray A., Han C., Bisignano C., Rao P., Wool E. (2022). Global burden of bacterial antimicrobial resistance in 2019: A systematic analysis. Lancet.

[2] Smith R., Coast J. (2013). The true cost of antimicrobial resistance. BMJ.

[3] O’Neill J. (2016). Tackling Drug-Resistant Infections Globally: Final Report and Recommendations: The Review on Antimicrobial Resistance. https://amr-review.org.

[4] Aldeyab M.A., López-Lozano J.M., Gould I.M., Babar Z.-U.-D. (2020). Global antibiotics use and resistance. Global Pharmaceutical Policy.

[5] World Health Organization (2014). Antimicrobial Resistance: Global Report on Surveillance.

[6] Jirjees F.J., Al-Obaidi H.J., Sartaj M., Conlon-Bingham G., Farren D., Scott M.G., Gould I.M., López-Lozano J.M., Aldeyab M.A. (2020). Antibiotic use and resistance in hospitals: Time-series analysis strategy for determining and prioritising interventions. Hosp. Pharm. Eur..

[7] Al-Taani G.M., Scott M., Farren D., Gilmore F., McCullagh B., Hibberd C., McCorry A., Versporten A., Goossens H., Zarb P. (2018). Longitudinal point prevalence survey of antibacterial use in Northern Ireland using the European Surveillance of Antimicrobial Consumption (ESAC) PPS and Global-PPS tool. Epidemiol. Infect..

[8] Laxminarayan R., Duse A., Wattal C., Zaidi A.K.M., Wertheim H.F.L., Sumpradit N., Vlieghe E., Hara G.L., Gould I.M., Goossens H. (2013). Antibiotic resistance—The need for global solutions. Lancet Infect. Dis..

[9] Tamma P.D., Cosgrove S.E. (2011). Antimicrobial stewardship. Infect. Dis. Clin. North Am..

[10] Kumar S., Little P., Britten N. (2003). Why do general practitioners prescribe antibiotics for sore throat? Grounded theory interview study. Br. Med. J..

[11] Teixeira Rodrigues A., Roque F., Falcão A., Figueiras A., Herdeiro M.T. (2013). Understanding physician antibiotic prescribing behaviour: A systematic review of qualitative studies. Int. J. Antimicrob. Agents.

[12] Md Rezal R.S., Hassali M.A., Alrasheedy A.A., Saleem F., Md Yusof F.A., Godman B. (2015). Physicians’ knowledge, perceptions and behaviour towards antibiotic prescribing: A systematic review of the literature. Expert Rev. Anti. Infect. Ther..

[13] Deuster S., Roten I., Muehlebach S. (2010). Implementation of treatment guidelines to support judicious use of antibiotic therapy. J. Clin. Pharm. Ther..

[14] McNulty C.A.M., Boyle P., Nichols T., Clappison P., Davey P. (2007). Don’t wear me out—The public’s knowledge of and attitudes to antibiotic use. J. Antimicrob. Chemother..

[15] Johnson L.S., MacDougall C., Trivedi K.K. (2016). The Legislative Momentum of Antimicrobial Stewardship: The US Perspective. Curr. Treat. Options Infect. Dis..

[16] Gruppen L.D., Mangrulkar R.S., Kolars J.C. (2012). The promise of competency-based education in the health professions for improving global health. Hum. Resour. Health.

[17] Davidson L.K. (2011). A 3-year experience implementing blended TBL: Active instructional methods can shift student attitudes to learning. Med. Teach..

[18] Courteille O., Fahlstedt M., Ho J., Hedman L., Fors U., von Holst H., Felländer-Tsai L., Möller H. (2018). Learning through a virtual patient vs. recorded lecture: A comparison of knowledge retention in a trauma case. Int. J. Med. Educ..

[19] McCoy L., Pettit R.K., Kellar C., Morgan C. (2018). Tracking Active Learning in the Medical School Curriculum: A Learning-Centered Approach. J. Med. Educ. Curric. Dev..

[20] MacDougall C., Schwartz B.S., Kim L., Nanamori M., Shekarchian S., Chin-Hong P.V. (2017). An Interprofessional Curriculum on Antimicrobial Stewardship Improves Knowledge and Attitudes Toward Appropriate Antimicrobial Use and Collaboration. Open Forum Infect. Dis..

[21] Bennardi M., Diviani N., Saletti P., Gamondi C., Stüssi G., Cinesi I., Rubinelli S. (2022). A qualitative analysis of educational, professional and socio-cultural issues affecting interprofessional collaboration in oncology palliative care. Patient Educ. Couns..

[22] Dellit T.H., Owens R.C., McGowan J.E., Gerding D.N., Weinstein R.A., Burke J.P., Huskins W.C., Paterson D.L., Fishman N.O., Carpenter C.F. (2007). Infectious Diseases Society of America and the Society for Healthcare Epidemiology of America guidelines for developing an institutional program to enhance antimicrobial stewardship. Clin. Infect. Dis..

[23] Barlam T.F., Cosgrove S.E., Abbo L.M., Macdougall C., Schuetz A.N., Septimus E.J., Srinivasan A., Dellit T.H., Falck-Ytter Y.T., Fishman N.O. (2016). Implementing an antibiotic stewardship program: Guidelines by the Infectious Diseases Society of America and the Society for Healthcare Epidemiology of America. Clin. Infect. Dis..

[24] Luther V.P., Ohl C.A., Hicks L.A. (2013). Antimicrobial stewardship education for medical students. Clin. Infect. Dis..

[25] Pulcini C., Wencker F., Frimodt-Møller N., Kern W.V., Nathwani D., Rodríguez-Baño J., Simonsen G.S., Vlahović-Palčevski V., Gyssens I.C., Jacobs F. (2015). European survey on principles of prudent antibiotic prescribing teaching in undergraduate students. Clin. Microbiol. Infect..

[26] Castro-Sánchez E., Drumright L.N., Gharbi M., Farrell S., Holmes A.H. (2016). Mapping antimicrobial stewardship in undergraduate medical, dental, pharmacy, nursing and veterinary education in the United Kingdom. PLoS ONE.

[27] Akande-Sholabi W., Ajamu A.T. (2021). Antimicrobial stewardship: Assessment of knowledge, awareness of antimicrobial resistance and appropriate antibiotic use among healthcare students in a Nigerian University. BMC Med. Educ..

[28] Sobierajski T., Mazińska B., Wanke-Rytt M., Hryniewicz W. (2021). Knowledge-based attitudes of medical students in antibiotic therapy and antibiotic resistance. A cross-sectional study. Int. J. Environ. Res. Public Health.

[29] Mudenda S., Mukela M., Matafwali S., Banda M., Mutati R.K., Muungo L.T., Daka V., Saad S.A.M., Bumbangi F.N., Chabalenge B. (2022). Knowledge, Attitudes, and Practices towards Antibiotic Use and Antimicrobial Resistance among Pharmacy Students at the University of Zambia: Implications for Antimicrobial Stewardship Programmes. Sch. Acad. J. Pharm..

[30] Ghadeer A.R.Y. (2012). Suaifan A cross-sectional study on knowledge, attitude and behavior related to\antibiotic use and resistance among medical and non-medical university students in Jordan. African J. Pharm. Pharmacol..

[31] Matalqah L.M., Albals D., Radaideh K.M., Al-Khateeb H., Thabet R.H., Abu-Ismail L., Abunasser M. (2022). Knowledge, Attitudes and Practice toward Antibiotic Use among Under and Post-Graduate Students at Yarmouk University in Jordan: A Descriptive Study. Jordan J. Pharm. Sci..

[32] Al-Qerem W., Hammad A., Jarab A., Saleh M.M., Amawi H.A., Ling J., Alasmari F. (2022). Knowledge, attitudes, and practice with respect to antibiotic use among pharmacy students: A cross-sectional study. Eur. Rev. Med. Pharmacol. Sci..

[33] Ashiru-Oredope D., Hopkins S., Vasandani S., Umoh E., Oloyede O., Nilsson A., Kinsman J., Elsert L., Monnet D.L. (2021). Healthcare workers’ knowledge, attitudes and behaviours with respect to antibiotics, antibiotic use and antibiotic resistance across 30 EU/EEA countries in 2019. Eurosurveillance.

[34] European Centre for Disease Prevention and Control (2019). Survey of Healthcare Workers’ Knowledge, Attitudes and Behaviours on Antibiotics, Antibiotic Use and Antibiotic Resistance in the EU/EEA.

[35] Brown E.M. (2002). Guidelines for antibiotic usage in hospitals. J. Antimicrob. Chemother..

[36] Sakeena M.H.F., Bennett A.A., Jamshed S., Mohamed F., Herath D.R., Gawarammana I., McLachlan A.J. (2018). Investigating knowledge regarding antibiotics and antimicrobial resistance among pharmacy students in Sri Lankan universities. BMC Infect. Dis..

[37] Inácio J., Barnes L.M., Jeffs S., Castanheira P., Wiseman M., Inácio S., Bowler L., Lansley A. (2017). Master of Pharmacy students’ knowledge and awareness of antibiotic use, resistance and stewardship. Curr. Pharm. Teach. Learn..

[38] Ahmad A., Khan M.U., Patel I., Maharaj S., Pandey S., Dhingra S. (2015). Knowledge, attitude and practice of B.Sc. Pharmacy students about antibiotics in Trinidad and Tobago. J. Res. Pharm. Pract..

[39] Higuita-Gutiérrez L.F., Villamil G.E.R., Quiceno J.N.J. (2020). Knowledge, attitude, and practice regarding antibiotic use and resistance among medical students in Colombia: A cross-sectional descriptive study. BMC Public Health.

[40] Afzal Khan A.K., Banu G., Reshma K.K. (2013). Antibiotic resistance and usage-a survey on the knowledge, attitude, perceptions and practices among the medical students of a southern Indian teaching hospital. J. Clin. Diagnostic Res..

[41] Abubakar U., Muhammad H.T., Sulaiman S.A.S., Ramatillah D.L., Amir O. (2020). Knowledge and self-confidence of antibiotic resistance, appropriate antibiotic therapy, and antibiotic stewardship among pharmacy undergraduate students in three Asian countries. Curr. Pharm. Teach. Learn..

[42] Ganesh M., Sridevi S.A., Paul C.M. (2014). Antibiotic use among Medical and Para Medical Students: Knowledge, Attitude and its Practice in a Tertiary Health Care Centre in Chennai—A Scientific Insight. Int. J. Sci. Res..

[43] Aveni E., Bauer B., Ramelet A.S., Decosterd I., Ballabeni P., Bonvin E., Rodondi P.Y. (2017). Healthcare professionals’ sources of knowledge of complementary medicine in an academic center. PLoS ONE.

[44] Mungrue K., Brown T., Hayes I., Ramroop S., Thurston P., Pinto Pereira L. (2009). Drugs in upper respiratory tract infections in paediatric patients in North Trinidad. Pharm. Pract..

[45] Thriemer K., Katuala Y., Batoko B., Alworonga J.P., Devlieger H., Van Geet C., Ngbonda D., Jacobs J. (2013). Antibiotic Prescribing in DR Congo: A Knowledge, Attitude and Practice Survey among Medical Doctors and Students. PLoS ONE.

[46] Ahmad A., Khan M.U., Moorthy J., Jamshed S.Q., Patel I. (2015). Comparison of knowledge and attitudes about antibiotics and resistance, and antibiotics selfpracticing between Bachelor of Pharmacy and Doctor of Pharmacy students in Southern India. Pharm. Pract..

[47] Wasserman S., Potgieter S., Shoul E., Constant D., Stewart A., Mendelson M., Boyles T.H. (2017). South African medical students’ perceptions and knowledge about antibiotic resistance and appropriate prescribing: Are we providing adequate training to future prescribers?. S. Afr. Med. J..

[48] Hoque R., Mostafa A., Haque M. (2016). Insight of medical students of clinical years to antimicrobials prescribing and resistance in private medical school, Chittagong, Bangladesh. J. Young Pharm..

[49] Oh A.L., Hassali M.A., Al-Haddad M.S., Sulaiman S.A.S., Shafie A.A., Awaisu A. (2011). Public knowledge and attitudes towards antibiotic usage: A cross-sectional study among the general public in the state of Penang, Malaysia. J. Infect. Dev. Ctries..

[50] Torres N.F., Chibi B., Middleton L.E., Solomon V.P., Mashamba-Thompson T.P. (2019). Evidence of factors influencing self-medication with antibiotics in low and middle-income countries: A systematic scoping review. Public Health.

[51] Abbo L.M., Cosgrove S.E., Pottinger P.S., Pereyra M., Sinkowitz-Cochran R., Srinivasan A., Webb D.J., Hooton T.M. (2013). Medical students’ perceptions and knowledge about antimicrobial stewardship: How are we educating our future prescribers?. Clin. Infect. Dis..

[52] Scaioli G., Gualano M.R., Gili R., Masucci S., Bert F., Siliquini R. (2015). Antibiotic Use: A cross-sectional survey assessing the knowledge, attitudes and practices amongst students of a school of medicine in Italy. PLoS ONE.

[53] Amann S., Neef K., Kohl S. (2019). Antimicrobial resistance (AMR). Eur. J. Hosp. Pharm. Sci. Pract..

[54] Justo J.A., Gauthier T.P., Scheetz M.H., Chahine E.B., Bookstaver P.B., Gallagher J.C., Hermsen E.D., DePestel D.D., Ernst E.J., Jacobs D.M. (2014). Knowledge and attitudes of doctor of pharmacy students regarding the appropriate use of antimicrobials. Clin. Infect. Dis..

[55] Minen M.T., Duquaine D., Marx M.A., Weiss D. (2010). A survey of knowledge, attitudes, and beliefs of medical students concerning antimicrobial use and resistance. Microb. Drug Resist..

[56] Zulu A., Matafwali S.K., Banda M., Mudenda S. (2020). Assessment of knowledge, attitude and practices on antibiotic resistance among undergraduate medical students in the school of medicine at the University of Zambia. Int. J. Basic Clin. Pharmacol..

[57] Silverberg S.L., Zannella V.E., Countryman D., Ayala A.P., Lenton E., Friesen F., Law M. (2017). A review of antimicrobial stewardship training in medical education. Int. J. Med. Educ..

[58] Fejza A., Kryeziu Z., Kadrija K., Musa M. (2016). Pharmacy students’ knowledge and attitudes about antibiotics in Kosovo. Pharm. Pract..

[59] Sarahroodi S., Arzi A., Sawalha A.F., Ashtarinezhad A. (2010). Antibiotics self-medication among southern iranian university students. Int. J. Pharmacol..

[60] Wright P.E., Jain P. (2004). Survey of antibiotic knowledge amongst final year medical students. J. Antimicrob. Chemother..

[61] Davey P., Marwick C.A., Scott C.L., Charani E., Mcneil K., Brown E., Gould I.M., Ramsay C.R., Michie S. (2017). Interventions to improve antibiotic prescribing practices for hospital inpatients. Cochrane Database Syst. Rev..

[62] Simpson S.A., Wood F., Butler C.C. (2007). General practitioners’ perceptions of antimicrobial resistance: A qualitative study. J. Antimicrob. Chemother..

[63] Roumie C.L., Halasa N.B., Edwards K.M., Zhu Y., Dittus R.S., Griffin M.R. (2005). Differences in antibiotic prescribing among physicians, residents, and nonphysician clinicians. Am. J. Med..

[64] Guerra C.M., Pereira C.A.P., Neto A.R.N., Cardo D.M., Correa L. (2007). Physicians’ Perceptions, Beliefs, Attitudes, and Knowledge Concerning Antimicrobial Resistance in a Brazilian Teaching Hospital. Infect. Control Hosp. Epidemiol..

[65] Schwartz B.S., Armstrong W.S., Ohl C.A., Luther V.P. (2015). Create Allies, IDSA Stewardship Commitments Should Prioritize Health Professions Learners. Clin. Infect. Dis..

[66] Paterson Davenport L.A., Davey P.G., Ker J.S., Finch R., Harden R., Holmes B., Leese J., MacGowan A., Masterton B., Morgan M. (2005). An outcome-based approach for teaching prudent antimicrobial prescribing to undergraduate medical students: Report of a Working Party of the British Society for Antimicrobial Chemotherapy. J. Antimicrob. Chemother..

[67] Ohl C.A., Luther V.P. (2014). Health care provider education as a tool to enhance antibiotic stewardship practices. Infect. Dis. Clin. North Am..

[68] Ntirenganya C., Manzi O., Muvunyi C.M., Ogbuagu O. (2015). High prevalence of antimicrobial resistance among common bacterial isolates in a tertiary healthcare facility in Rwanda. Am. J. Trop. Med. Hyg..

[69] Chen C., Chen Y.M., Hwang K.L., Lin S.J., Yang C.C., Tsay R.W., Liu C.E., Young T.G. (2005). Behavior, attitudes and knowledge about antibiotic usage among residents of Changhua, Taiwan. J. Microbiol. Immunol. Infect..

[70] Srinivasan A., Song X., Richards A., Sinkowitz-Cochran R., Cardo D., Rand C. (2004). A survey of knowledge, attitudes, and beliefs of house staff physicians from various specialties concerning antimicrobial use and resistance. Arch. Intern. Med..

[71] Nisabwe L., Brice H., Umuhire M.C., Gwira O., Harelimana J.D.D., Nzeyimana Z., Sebatunzi O.R., Rusingiza E.K., Hahirwa I., Muvunyi C.M. (2020). Knowledge and attitudes towards antibiotic use and resistance among undergraduate healthcare students at University of Rwanda. J. Pharm. Policy Pract..

[72] Shapiro D.J., Hicks L.A., Pavia A.T., Hersh A.L. (2014). Antibiotic prescribing for adults in ambulatory care in the USA, 2007–2009. J. Antimicrob. Chemother..

[73] Ministry of Health National Action Plan for Combating Antimicrobial Resistance in the Hashemite Kingdom of Jordan. https://cdn.who.int/media/docs/default-source/antimicrobial-resistance/amr-spc-npm/nap-library/jordan_amr-nap_2018-2022.pdf?sfvrsn=83be0540_1&download=true.

[74] Huang Y., Gu J., Zhang M., Ren Z., Yang W., Chen Y., Fu Y., Chen X., Cals J.W., Zhang F. (2013). Knowledge, attitude and practice of antibiotics: A questionnaire study among 2500 Chinese students. BMC Med. Educ..

[75] Charani E., Castro-Sanchez E., Sevdalis N., Kyratsis Y., Drumright L., Shah N., Holmes A. (2013). Understanding the determinants of antimicrobial prescribing within hospitals: The role of “prescribing etiquette”. Clin. Infect. Dis..

[76] Mattick K., Kelly N., Rees C. (2014). A window into the lives of junior doctors: Narrative interviews exploring antimicrobial prescribing experiences. J. Antimicrob. Chemother..

[77] De Souza V., MacFarlane A., Murphy A.W., Hanahoe B., Barber A., Cormican M. (2006). A qualitative study of factors influencing antimicrobial prescribing by non-consultant hospital doctors. J. Antimicrob. Chemother..

[78] World Health Organization WHO Global Strategy for Containment of Antimicrobial Resistance. https://apps.who.int/iris/bitstream/handle/10665/66860/WHO_CDS_CSR_DRS_2001.2.pdf.

[79] (2015). Resolution WHA 68-7 Global Action Plan on Antimicrobial Resistance. Microbe Mag..

[80] WHO Regional Office for Europe (2014). The Role of Pharmacist in Encouraging Prudent Use of Antibiotics and Averting Antimicrobial Resistance: A Review of Policy and Experience.

[81] Southwick F., Katona P., Kauffman C., Monroe S., Pirofski L.A., Del Rio C., Gallis H., Dismukes W. (2010). Commentary: IDSA guidelines for improving the teaching of preclinical medical microbiology and infectious diseases. Acad. Med..

[82] Shekarchian S., Schwartz B.S., Teherani A., Irby D., Chin-Hong P.V. (2016). Is It Time for a Coordinated and Longitudinal Approach to Antibiotic Stewardship Education?. Clin. Infect. Dis..

[83] Fishman N. (2012). Policy Statement on Antimicrobial Stewardship by the Society for Healthcare Epidemiology of America (SHEA), the Infectious Diseases Society of America (IDSA), and the Pediatric Infectious Diseases Society (PIDS). Infect. Control Hosp. Epidemiol..

[84] Pulcini C., Gyssens I.C. (2013). How to educate prescribers in antimicrobial stewardship practices. Virulence.

[85] Lee C.-R., Lee J.H., Kang L.-W., Jeong B.C., Lee S.H. (2015). Educational Effectiveness, Target, and Content for Prudent Antibiotic Use. Biomed Res. Int..

[86] Majumder M.A.A., Singh K., Hilaire M.G.S., Rahman S., Sa B., Haque M. (2020). Antimicrobial resistance and global public health crisis: Are we educating enough our future prescribers to combat this global threat?. Anti. Infect. Ther..

[87] Dyar O.J., Pulcini C., Howard P., Nathwani D., Beovic B., Harbarth S., Hanberger H., Pagani L., Pardo J.R.P., Weschesler-Fördös A. (2014). European medical students: A first multicentre study of knowledge, attitudes and perceptions of antibiotic prescribing and antibiotic resistance. J. Antimicrob. Chemother..

