# Supplementary material for: Knowledge, Attitude, and Behavior about Antimicrobial Use and Resistance among Medical, Nursing and Pharmacy Students in Jordan: A Cross Sectional Study
Source: Antibiotics (Basel). 2022 Nov 5;11(11):1559. doi: 10.3390/antibiotics11111559 (PMC9686822; doi:10.3390/antibiotics11111559)
Supplement: Supplementary file 1 [file antibiotics-11-01559-s001.zip › antibiotics-1942898-supplementary.pdf]

Survey of students of health sciencesknowledge and attitudes about antibiotics and antibiotic resistance

Dear Student,

You are invited to complete the following survey about students' knowledge and attitudes about antibiotics and antibiotic resistance.

Researchers from UK, Jordan University of Science and Technology, and Yarmouk University are seeking responses from students. We would really value you completing the survey that will take 10 to 15 minutes to complete. It includes predominantly multiple choice questions.

Please feel free to cascade the link of the survey widely to colleagues.

* Required

Demographic Section

1. Please specify in which governorate you live: *

- Amman
- Irbid
- Salt
- Zarqa
- Madaba
- Jeras
- Ajloun
- Mafraq
- Karak
- Tafilah
- Ma'an
- Aqaba

2. What career are you currently studying for? *

- Medicine
- Nursing

- Pharmacy
- Dentistry
- Scientist
- Other: ____________________________

3. What year of your studies are you in? *

- 1 st year
- 2nd year
- 3rd year
- 4th year
- 5th year or later
- Other: _____________________________

4. What university are you studying at: *

____

5. What is your age? *

- 18-25 years
- 26-35 years
- 36-45 years
- 46-55 years
- 56-65 years
- >66 years

6. What gender do you identify with? *

- Male
- Female

7. Which of the following social media networks do you mainly use for professionalactivities? *

Check all that apply.

- ☐ Twitter
- ☐ Facebook
- ☐ LinkedIn

- ☐ Google+
- ☐ YouTube
- ☐ Instagram
- ☐ I do not use social media
- ☐ Other:____________________________.

Information about antibiotic use and antibiotic resistance

8. Please answer whether you believe these statements are true or false. *

	True	False	Unsure
Antibiotics are effective against viruses			
**Antibiotics** are effective against cold and flu			
Unnecessary use of antibiotics makes thembecome ineffectiv			
**Taking antibiotics has** associated side effects or risks such as Diarrhoea, **colitis.**Allergies.			
Every person treated with antibiotics is at an increased risk of antibiotic resistant infection			
Antibiotic resistance bacteria can spread fromperson to persor			
Heathy people can carry antibiotic resistantbacteria			
The use **of antibiotics to** stimulate growth infarm animals is legal in Jordan			

9. In the management of infections, which of these do you use regularly? *

Check all that apply.

- ☐ Clinical practice guidelines
- ☐ Documentation from the pharmaceutical industry
- ☐ Medical representatives from industry
- ☐ Previous clinical experience
- ☐ Continuing education training courses
- ☐ Infection specialists
- ☐ Scientific journals
- ☐ Professional resources/publications

- ☐ Social media
- ☐ None of the above
- ☐ I do not know
- ☐ Other: ___________________

Campaign and Training questions

10. At what level do you think it is most effective to tackle resistance to antibiotics?Select no more than 2. *

Check all that apply.

- ☐ Individual level (prescribers)
- ☐ Environmental/Animal Health
- ☐ Regional/National Level Global
- ☐ Action at all levels needed
- ☐ I do not know

11. What initiatives are you aware of in your country which focus on antibioticawareness and resistance? Select all that apply *

Check *all that apply.*

- ☐ TV or Radio advertising for the public
- ☐ Toolkits and resources for healthcare workers
- ☐ National or regional guidelines on management of infections
- ☐ Awareness raising from professional organisations
- ☐ Conference/Events focused on tackling antibiotic resistance
- ☐ National or regional posters or leaflets on antibiotic awareness
- ☐ Newspaper (national) articles on antibiotic resistance
- ☐ World Antibiotic Awareness Week
- ☐ I am not aware of any initiatives
- ☐ Other:________________

12. Regarding the national initiatives about prudent use of antibiotics in your country, to what extent do you agree or disagree with this statements "There has been good promotion of prudent use of antibiotics and antibiotic resistance in mycountry"? *

- Strongly Disagree
- Disagree
- Undecided
- Agree
- Strongly Agree
- I do not remember
- Not applicable

13. Does your country have a national action plan on antimicrobial resistance *

- Yes
- No
- Unsure

14. Have you had any teaching about antibiotic treatment and prudent antibiotic use during your undergraduate degree? *

	Yes	No	Unsure
Prudent antibiotic use			
Management of infections (diagnosis andantibiotic treatment)			

15. Have any of your examinations included questions about antibiotic treatment or prudent use of antibiotics? *

	Yes	No	Unsure
Prudent antibiotic use			
Management of infections (diagnosis andantibiotic treatment)			

16. On which topics would you like to receive more information? *

Check all that apply.

- ☐ Resistance to antibiotics
- ☐ How to use antibiotics
- ☐ Medical conditions for which antibiotics are used
- ☐ Prescription of antibiotics
- ☐ Links between the health of humans, animals and the environment
- ☐ None
- ☐ Other:______________________

Which of the following methods of teaching have been used to teach you about prudent use of antibiotics/antibiotic treatment and how useful would you rate them?

17. Lectures (with >15 people): *

- Not very useful
- Not useful
- Undecided
- Useful
- Very useful
- I do not understand the question
- Not applicable

18. Small group teaching (with <15 people): *

- Not very useful
- Not useful
- Undecided
- Useful
- Very useful
- I do not understand the question
- Not applicable

19. Discussions of clinical cases and vignettes: *

- Not very useful
- Not useful
- Undecided
- Useful
- Very useful
- I do not understand the question
- Not applicable

20. Active learning assignments (e.g. article reading, group work, preparing an oralpresentation):

- Not very useful
- Not useful
- Undecided
- Useful
- Very useful
- I do not understand the question
- Not applicable

21. E-learning: *

- Not very useful
- Not useful
- Undecided
- Useful
- Very useful
- I do not understand the question
- Not applicable

22. Role play or communication skills sessions dealing with patients demandingantibiotic training:

- Not very useful
- Not useful
- Undecided
- Useful
- Very useful
- I do not understand the question

- Not applicable

23. Infectious diseases clinical placement (i.e. clinical rotation or training ininfectious diseases, involving patients): *

- Not very useful
- Not useful
- Undecided
- Useful
- Very useful
- I do not understand the question
- Not applicable

24. Microbiology clinical placement: *

- Not very useful
- Not useful
- Undecided
- Useful
- Very useful
- I do not understand the question
- Not applicable

25. Peer or near peer- teaching (i.e.teaching led by other students or recentlyqualified doctors): *

- Not very useful
- Not useful
- Undecided
- Useful
- Very useful
- I do not understand the question
- Not applicable

For the next questions, to what extent do you agree or disagree with the followingstatements:

26."I know what antibiotic resistance is" *

- Strongly disagree
- Disagree Undecided
- Agree
- Strongly Agree
- I do not understand the question
- Not Applicable

27. "I know there is a connection between my prescribing OR dispensing OR administering of antibiotics and emergence and spread of antibiotic resistant bacteria": *

- Strongly disagree
- Disagree Undecided
- Agree
- Strongly Agree
- I do not understand the question
- Not Applicable

28."I know what information to give to individuals about prudent use of antibioticsand antibiotic resistance": *

- Strongly disagree
- Disagree Undecided
- Agree
- Strongly Agree
- I do not understand the question
- Not Applicable

29. "I have sufficient knowledge about how to use antibiotics appropriately for mycurrent practice": *

- Strongly disagree
- Disagree Undecided
- Agree

- Strongly Agree
- I do not understand the question
- Not Applicable

30. "I have a key role in helping control antibiotic resistance'. *

- Strongly disagree
- Disagree Undecided
- Agree
- Strongly Agree
- I do not understand the question
- Not Applicable

31. "I have easy access to guidelines I need on managing infections": *

- Strongly disagree
- Disagree Undecided
- Agree
- Strongly Agree
- I do not understand the question
- Not Applicable

32. "I have easy access to the materials I need to give advice on prudent antibiotic use and antibiotic resistance": *

- Strongly disagree
- Disagree Undecided
- Agree
- Strongly Agree
- I do not understand the question
- Not Applicable

33. "I have good opportunities to provide advice on prudent antibiotic use toindividuals": *

- Strongly disagree
- Disagree Undecided
- Agree

- Strongly Agree
- I do not understand the question
- Not Applicable

34. "Environmental factors such as waste water in the environment are important in contributing to antibiotic resistance in bacteria from humans"? *

- Strongly disagree
- Disagree Undecided
- Agree
- Strongly Agree
- I do not understand the question
- Not Applicable

35. "Excessive use of antibiotics in livestock and food production is important incontributing to antibiotic resistance in bacteria from humans"? *

- Strongly disagree
- Disagree Undecided
- Agree
- Strongly Agree
- I do not understand the question
- Not Applicable

Please answer the following questions, considering the last one week only in yourclinical practice:

36. How often did you give out resources (e.g. leaflets or pamphlets) on prudent antibiotic use or management of infections to individuals during the last one week?

- Once a day
- More than once a day
- Once a week
- More than once a week
- rarely
- never

- I do not remember
- Not Applicable

37. How often did you give out advice related to prudent antibiotic use or management of infections to an individual during the last one week? *

- Once a day
- More than once a day
- Once a week
- More than once a week
- rarely
- never
- I do not remember
- Not Applicable
